# Evaluating the association between gingival crevicular blood glucose levels and finger capillary blood glucose levels according to periodontal status

**DOI:** 10.1186/s40001-023-01611-8

**Published:** 2024-01-30

**Authors:** Somaye Ansari Moghadam, Hanie Abbaszade, Mohadeseh Sartipi, Alireza Ansari Moghadam

**Affiliations:** 1 Department of Periodontology, Oral and Dental Disease Research Center, Zahedan University of Medical Sciences, Zahedan, Iran; 2 Oral and Dental Disease Research Center, Zahedan University of Medical Sciences, Zahedan, Iran; 3 Sri Rajiv Gandhi College of Dental Science & Hospital, Rajiv Gandhi University of Health Sciences, Bangalore, India; 4 Department of Health, Promotion Research Center, Zahedan University of Medical Sciences, Zahedan, Iran

**Keywords:** Gingival crevicular blood glucose, Capillary blood glucose, Periodontal disease, Diabetes mellitus

## Abstract

**Objectives:**

The aim of this study was to evaluate the association between gingival crevicular blood glucose levels (GCBG) and finger capillary blood glucose levels (FCBG) according to the periodontal status of patients.

**Materials and methods:**

In this case–control study, 80 patients were divided into 4 groups according to their periodontal status. In these patients, an area of the maxillary gingiva with the highest probing depth was selected for blood sampling. Blood glucose obtained from this area and the right fingertip was measured with a glucometer. Data were analyzed using ANOVA and Pearson correlation coefficient with a significance threshold of 0.05.

**Results:**

The groups studied were matched in regard to their sex and age (*P* > 0.05). The average FCBG and GCBG were not significantly different according to periodontal status (*P* > 0.05). The correlation between the FCBG and the GCBG showed a significant positive correlation in the total number of participants (*P* < 0.05, *r* = 0.531).

**Conclusion:**

The study observed a positive association between GCBG and FCBG. However, the relationship with periodontal status appeared to be relatively weak. Further research may be needed to determine the potential efficacy of GCBG in diabetes screening during periodontal examinations. Clinical relevance: Most patients with diabetes do not have proper periodontal health, so it may be helpful to screen for diabetes during periodontal examinations.

## Background

Globally, there has been a steady increase in diabetic mellitus, a metabolic disease affecting insulin production [1, 2]. Notably, periodontitis patients are more than twice as likely to have diabetes compared to their healthy counterparts [1], highlighting the bidirectional relationship between these two common chronic diseases [3]. Moreover, periodontal treatment can have a potentially positive impact on glycosylated hemoglobin (HbA1c) levels, thereby improving metabolic control [4].

Various aspects of the periodontium are affected by diabetes including subgingival microbiota, gingival blood glucose levels, periodontal blood vessels, host defenses, and collagen metabolism. Meanwhile, periodontal disease, the sixth most common complication of diabetes [1], often manifests with symptoms such as bleeding gums, periodontal pockets, and alveolar bone destruction in most adult patients [5, 6].

Controlling diabetes in patients with Stage 3 Grade C periodontitis can be challenging and ineffective. However, by managing and treating periodontal disease, diabetes can be effectively controlled [7, 8]. Periodontists normally rely on laboratory tests, using intravenous or capillary blood, to assess patients’ blood glucose control, which may not reflect their current status [1].

Several studies have investigated the correlation between gingival crevicular blood glucose (GCBG) and other blood glucose measurements. There have been some contradictory findings with some studies reporting strong positive correlations, while others finding less significant correlations.

A 2020 study by Saeed et al. showed a strong positive correlation between GCBG and FCBG levels and a favorable correlation between GCBG and hemoglobin A1c levels [3]. Rapone et al. also verified a strong correlation between fasting capillary blood glucose (FCBG) and gingival crevicular blood glucose (GCBG) levels in both healthy and diabetic individuals [9].

Previously, Singh et al. in 2019 showed a strong correlation between GCBG and capillary blood glucose (CBG) as well as GCBG and plasma glucose levels [10]. Nayagam et al. demonstrated a strong positive correlation between gingival crevicular blood glucose (GCBG) and capillary blood glucose levels in both gingivitis and periodontitis patients [11]. Arora et al. also confirmed their findings by displaying an excellent positive correlation between GCBG and FCBG [12].

However, the following three studies reached different conclusions. In 2017, Subodh et al. found no statistically significant difference between GCBG and fasting capillary blood glucose (FCBG) levels in diabetic and nondiabetic patients [13]. Similarly, in a 2014 study, Kandwal et al. failed to validate the use of GCBG for blood glucose testing during routine periodontal examinations, both in diabetic and nondiabetic subjects [14].

Moreover, even within the same study, varying conclusions were drawn; a 2011 study by Waghmare et al. examined patients with varying degrees of bleeding during probing and identified a strong correlation between GCBG and capillary blood glucose in group A (those with heavy bleeding), while the correlation in group B (those with slight bleeding) was weak [15].

In response to the discrepancies between the previous studies, our study aimed to investigate the association between FCBG and GCBG according to the patient’s periodontal status, to introduce a noninvasive method for diabetes detection in dental clinics.

## Material and methods

The present case‒control study started after receiving permission from the ethics committee of the Zahedan University of Medical Sciences with the ethical code IR.ZAUMS.REC.1399.311.

Eighty patients referred to the Zahedan Dental School’s Periodontal Department who met the following inclusion criteria were selected. After obtaining the patient’s demographic information, periodontal examinations were performed. The inclusion criteria included the following:

Patients over 20-year old [2], nonsmoker and nonusers of other tobacco products, not pregnant or breastfeeding, not taking systemic antibiotics [2], no previous periodontal treatments in the last 6 months [11], have at least 20 teeth present, absence of bleeding disorders, absence of systemic diseases (other than diabetes mellitus) affecting periodontal conditions, did not use drugs that interfere with the coagulation system [2], and presence of active inflammation in periodontal tissue (bleeding during probing) [11].

In the present study, age and sex were matched. The periodontal status of the patients was determined based on the new classification scheme put forth by Caton et al. [16], and each patient was placed in one of the following four groups: gingivitis, Stage 1 Grade A, Stage 2 Grade B, and Stage 3 Grade C, periodontitis.

The examiner (H. A), with an inter-operator kappa agreement of 0.9, used an HUE-FRIEDY Williams periodontal probe (1 mm accuracy) to measure GCBG. To maintain proper control, we focused solely on one specific oral cavity region, selecting the maxillary anterior area with the deepest probe depth that was also BOP positive. After determining the area, the first drop of blood seeping from the gingival sulcus was flushed to minimize contamination. After isolating the site with cotton, gauze, or compressed air, we used a Williams probe to stimulate blood in the gingival sulcus. At this stage, 2 to 3 mL of gingival crevicular blood was collected by a micropipette and then transferred to a special test area (strip test). Then, it was measured and recorded with a glucometer (glucocard, 0.1 ARKRAY manufacturer, made in Japan) with 99% accuracy. Gingival crevicular blood glucose in mg/dL was displayed on the glucometer display [11]. The glucometer was calibrated before the measurements. To measure capillary blood glucose of the nondominant fingertip (index finger), the patient was first asked to wash and dry the nondominant fingertip with clean soap and water. After this step, the index finger was pierced using a sterile lancet, and the first drop formed was discarded. The second drop of blood was transferred to a special area for measurement (strip test). It was then transferred to a glucometer, and its data were recorded. The unit mg/dL was presented on the glucometer display [11].

If the blood sample collected for measuring blood glucose was not suitable, the sample was excluded from the study.

By explaining the stages of the study, informed consent was obtained from the patients.

They were also taught how to brush, floss, and use mouthwash properly. After that, if necessary, scaling and root planing were performed.

To describe the data, descriptive statistics, including statistical tables, means, and standard deviations, were used. To compare the averages, one-way ANOVA testing was used, and to evaluate the correlation between average GCBG and FCBG, the Pearson correlation test, according to the normal distribution of data, was used. The significance level was considered to be 0.05. Data were analyzed using SPSS software version 24.

## Results

In this study, 80 patients referred to the Department of Periodontics, Zahedan Dental School, were studied.

47.5% (38 people) of the participants were female and 52.5% (42 people) were male.

The average age of male participants was 25.81 ± 6.39 (age range 18 to 55 years), and the average age of female participants was 30.30 ± 7.56 (age range 21 to 52 years).

The normality of the data in the studied groups (gingivitis, Stage 1 Grade A, Stage 2 Grade B and Stage 3 Grade C periodontitis) is investigated in Table 1.

**Table 1:** Evaluation of data normality by the Kolmogorov‒Smirnov test

Periodontal status	Variable	Kolmogotov–Smirnov	
		*P* value	*Z* score
Gingivitis	Capillary blood glucose	0.418	0.882
	Gingival crevicular blood glucose	0.122	1.182
	Age	0.790	0.651
Stage 1 Grade A	Capillary blood glucose	0.741	0.685
	Gingival crevicular blood glucose	0.621	0.754
	Age	0.161	1.122
Stage 2 Grade B	Capillary blood glucose	0.344	0.683
	Gingival crevicular blood glucose	0.739	0.936
	Age	0.434	0.871
Stage 3 Grade C	Capillary blood glucose	0.238	1.032
	Gingival crevicular blood glucose	0.867	0.598
	Age	0.471	0.846

The normality of the data in the studied groups (gingivitis, Stage 1 Grade A, Stage 2, Grade B Stage 2 Grade B and Stage 3 Grade C periodontitis) was investigated in Table 4

*P* value from Kolmogotov–Smirnov

According to Table 1, the normality of capillary blood glucose, gingival crevicular blood glucose, and age was evaluated separately for each study group using the Kolmogorov‒Smirnov test, and it was found that all data followed a normal distribution (*P* > 0.05). Therefore, parametric tests were used to compare the averages and correlations.

According to Table 2, based on the chi-square test, the studied groups were not significantly different according to sex, so the study groups were homogeneous according to sex (*P* > 0.05).

**Table 2:** Frequency distribution of study participants by group and gender

Gender	Groups								Total	Chi-square test	
	Gingivitis		Stage 1 Grade A		Stage 2 Grade B		Stage 3 Grade C			*P* value	Value
	Number of patients	Percentage	Number of patients	Percentage	Number of patients	Percentage	Number of patients	Percentage			
Female	7	35	12	60	11	55	8	40	[47.538]	3.409	0.334
Male	13	65	8	40	9	45	12	60	[52.542]		
Total	20	100	20	100	20	100	20	100	80		

*P* value from Chi-square test

According to Table 3 and based on one-way ANOVA test, the average age was not statistically significant between the groups of gingivitis, Stage 1 Grade A, Stage 2 Grade B and Stage 3 Grade C periodontitis (*P* > 0.05).

**Table 3:** Assessing the average age of the subjects based on periodontal stage

Periodontal stage	Number of patients	Average	Standard deviation	95% certainty		*P* value
				Lower limit	Upper limit	
Gingivitis	20	26.95	8.05	23.17	30.72	0.787
Stage 1 Grade A	20	28.90	7.43	25.42	32.37	
Stage 2 Grade B	20	29.05	6.84	25.84	32.25	
Stage 3 Grade C	20	27.80	7.36	24.35	31.24	
Total	80	28.17	7.34	26.54	29.80	

*P* value from one-way ANOVA test

According to Table 4, the mean Finger capillary blood glucose was not statistically significant between the groups of gingivitis, Stage 1 Grade A, Stage 2 Grade B and Stage 3 Grade C periodontitis (*P* > 0.05).

**Table 4:** Determining the average finger capillary blood glucose based on periodontal status

Periodontal status	Number of patients	Average	Standard deviation	95% certainty		*P* value
				Lower limit	Upper limit	
Gingivitis	20	93.00	18.53	84.32	101.67	0.321
Stage 1 Grade A	20	102.45	16.98	94.50	110.39	
Stage 2 Grade B	20	96.50	18.82	87.69	105.30	
Stage 3 Grade C	20	95.50	11.30	89.76	100.33	
Total	80	96.75	16.74	93.02	100.47	

*P* value from one-way ANOVA test

According to Table 5, the average gingival crevicular blood glucose between the groups of patients with gingivitis, Stage 1 Grade A, Stage 2 Grade B and Stage 3 Grade C periodontitis was not statistically significant (*P* > 0.05).

**Table 5:** Determining the average gingival crevicular blood glucose based on periodontal status

Periodontal status	Number of patients	Average	Standard deviation	95% certainty		*P* value
				Lower limit	Upper limit	
Gingivitis	20	73.50	29.44	59.71	87.28	0.718
Stage 1 Grade A	20	63.45	33.46	47.78	79.11	
Stage 2 Grade B	20	65.55	30.44	51.30	79.79	
Stage 3 Grade C	20	69.90	25.91	57.77	82.02	
Total	80	68.10	29.62	61.50	74.69	

*P* value from one-way ANOVA test

According to Table 6, there is a significant positive correlation between average capillary blood glucose and GCBG; when one increases, the other also increases (*r* = 0.531 and *P* < 0.00).

**Table 6:** Determining the correlation between average gingival crevicular blood glucose and finger capillary blood glucose regardless of periodontal status

Blood glucose		Correlation coefficient	*P* value
Capillary	Gingival sulcus	<0.001	0.531

Pearson correlation coefficient

According to Table 7, there was a statistically significant positive correlation between the mean capillary blood glucose and the mean gingival crevicular blood glucose in the gingivitis (*P* < 0.05, *r* = 0.724), Stage 2 Grade B (*P* < 0.05, *r* = 0.802), and Stage 3 Grade C periodontitis (*P* < 0.05, *r* = 0.522) groups. However, there was no significant correlation between mean capillary blood glucose and mean gingival crevicular blood glucose in the group of patients with Stage 1 Grade A (*P* > 0.05, *r* = 0.246).

**Table 7:** Comparison of gingival crevicular blood glucose correlation with finger capillary blood glucose based on periodontal status

Periodontal status	Pearson correlation coefficient	*P* value
Gingivitis	< 0.001	0.724
Stage 1 Grade A	0.296	0.246
Stage 2 Grade B	< 0.001	0.802
Stage 3 Grade C	0.018	0.522

Pearson correlation coefficient

## Discussion

Diabetes and periodontitis have a complex bidirectional relationship which highlights the significance of these two prevalent diseases. Individuals with periodontal disease have a significantly higher likelihood of developing diabetes. With a notable presence of undiagnosed, asymptomatic diabetics among patients seeking dental care [17], early detection of this disease is very important due to its multifactorial nature. Therefore, screening for diabetes is the most important step in monitoring and managing the risks of this disease [9].

In general, periodontal diseases stand out as one of the most common diseases among humans [10]. Interestingly, there is a high probability that most dental patients may have never undergone diabetes testing, creating a good opportunity for blood glucose assessment within the dental clinic [3].

Standard methods for assessing blood glucose, involving capillary blood glucose levels from fingertip sampling, are usually inconvenient, leading to reduced patient participation [18, 19]. Therefore, it is better to look for more appropriate methods for blood glucose measurement.

Bleeding from the gums during probing (BOP), one of the most common symptoms of periodontal inflammation [20], provides an opportunity to examine the relationship between GCBG and FCBG in order to determine the reliability of the GCBG assessment method. In this study, a glucometer was used to measure patient blood samples with varying stages of periodontal disease (gingivitis, Stage 1 Grade A, Stage 2 Grade B, and Stage 3 Grade C, periodontitis).

In this study, the average GCBG levels across different periodontal status revealed no significant differences, thus suggesting that periodontal status does not have a noticeable impact on GCBG levels. These findings were consistent with those of Arora et al. [12] and Ardakani et al., who examined GCBG in people with gingivitis and periodontitis and did not find a significant difference between the GCBG levels of the two groups. Ardakani et al. [21], in a similar study, did not observe a difference between patients with stage 2 grade B, and stage 3 grade C, periodontitis in terms of GCBG levels. The results of these studies were consistent with the present study.

FCBG levels were also measured in participants in this study. After comparing FCBG values, no significant difference was found based on periodontal status. Studies by Arora et al. [12] and Ardakani et al. [21] also confirmed that FCBG is not affected by periodontal status.

The FCBG values obtained in this study were higher than the GCBG values in each periodontal group and in total. Studies by Singh et al. [10], Parihar et al. [22], Kaur et al. [26], and Waghmare et al. [15] also reported higher values for FCBG than GCBG.

Upon calculating the average FCBG and GCBG of all participants and within different periodontal health states, a positive yet weak association was observed, with an ‘*r*’ value of 0.531. These findings underline the difficulty of utilizing GCBG as a substitute for FCBG in diabetes screening during routine periodontal examinations. Numerous studies have examined this correlation [9, 14, 23, 24], and similar to the present study, they did not consider GCBG applicable for diabetes screening.

Conversely, many studies, in contradiction to our results, have detected a strong correlation between FCBG and GCBG. For example, Rapone et al. [9], who examined the correlation in people with a positive BOP, found this correlation convincing in those with diabetes. Waghmare et al. [15] also found that the above correlation was acceptable in people with heavy bleeding gums. In the studies of Saeed et al. [3], Singh et al. [10], Nayagam et al. [11], Sibyl et al. [25], Subodh et al. [13], Kaur et al. [26], Ardakani et al. [21], and Beikler et al. [27], all participants had stage 2 grade B to stage 3 grade C periodontitis and included patients diagnosed with diabetes. These studies also showed higher correlation values in the diabetic group than in the nondiabetic group. The increased severity of periodontal disease and the definite presence of diabetes may have increased the correlation between GCBG and FCBG. Hence, the conflicting findings of the present studies with the above studies can imply that the degree of the correlation is influenced by factors such as the severity of periodontal disease and the presence or absence of diabetes. The absence of diabetic patients in this study may explain the relatively weak correlation observed. Nonetheless, the theory of an increased correlation with worsening periodontal disease does not seem satisfactory, as suggested by the higher correlation values observed in individuals with gingivitis than in those with stage 1 grade A or stage 3 grade C periodontitis. Notably, variations in blood sample concentrations, glucometer type, sampling methods, and sample size variations among studies may contribute to differences in outcomes.

In the present study, sampling of the gingival sulcus area was performed using a micropipette after washing and complete isolation of the area to remove contaminants. This methodology was similar to the approach used in the studies by Ardakani and Parker, where a plastic pipette was used for sampling [21, 28].

In addition, it is worth noting that this study employed a new-generation glucometer, the glucocard device from ARKRAY Japan, which requires minimal blood volume (0.3 µL) to determine blood glucose levels. Unlike its predecessors, this device does not require manual adjustments and offers a high level of accuracy [23]. This device was not used in any of the previous studies, making our present study unique in this regard.

It is vital to recognize certain limitations that may have affected our study’s results. First, the small sample size raises concerns about statistical significance and generalizability. Moreover, our relatively condensed inclusion and exclusion criteria amassed from a variety of different studies could introduce confounding variables and selection bias. These limitations highlight the need for future research with larger samples and more developed criteria.

The results of the present study refuted the claim that GCBG can be used to screen for diabetes during routine periodontal examinations. Therefore, GCBG cannot be considered equivalent to FCBG. However, definitive confirmation of this theory requires further future studies. It is important to note that our research design and statistical tests were not meant to establish a direct correlation or causation.

According to this study, there is an association between gingival crevicular blood glucose and finger capillary blood glucose. Nevertheless, its association is weak, so it is not feasible to use GCBG instead of FCBG in diabetes screening during routine periodontal examination.

## Data Availability

The authors declare that the datasets used and/or analyzed during the current study are available from the corresponding author upon reasonable request.
